# Efficacy of Combined Encorafenib and Binimetinib Treatment for Erdheim–Chester Disease Harboring Concurrent *BRAF* ^V600E^ and *KRAS* ^G12R^ Mutations: A Case Report

**DOI:** 10.1002/cnr2.70093

**Published:** 2024-12-26

**Authors:** Yuto Hibino, Rika Sakai, Hiroyuki Takahashi, Takaaki Takeda, Natsuki Hirose, Mayumi Tokunaga, Kota Washimi, Tomoyuki Yokose, Rika Kasajima, Yukihiko Hiroshima, Yohei Miyagi, Hideaki Nakajima

**Affiliations:** ^1^ Department of Hematology and Medical Oncology Kanagawa Cancer Center Yokohama Japan; ^2^ Department of Pathology Kanagawa Cancer Center Yokohama Japan; ^3^ Center for Cancer Genome Medicine Kanagawa Cancer Center Yokohama Japan; ^4^ Kanagawa Cancer Center Research Institute Yokohama Japan; ^5^ Department of Hematology and Clinical Immunology Yokohama City University Graduate School of Medicine Yokohama Japan

**Keywords:** BRAF inhibitor, Erdheim–Chester disease, genomic profiling, histiocytosis, MEK inhibitor, molecular‐targeted therapy

## Abstract

**Background:**

Erdheim–Chester disease (ECD) is a rare form of non‐Langerhans cell histiocytosis with diverse clinical manifestations, often associated with mutations in the mitogen‐activated protein kinase/extracellular signal‐regulated kinase (MAPK/ERK) pathway. *BRAF* and *KRAS* mutations, which are driver mutations of oncogenes, participate in the same signaling pathway (MAPK/ERK pathway) and are usually mutually exclusive. We report a case of ECD with concurrent *BRAF* ^V600E^ and *KRAS* ^G12R^ mutations treated using BRAF and MEK inhibitors.

**Case:**

A 70‐year‐old man was referred to our hospital with a mesenteric nodal lesion on computed tomography scan. The patient experienced symptoms consistent with ECD, including central diabetes insipidus. Biopsy revealed histiocytes positive for CD68 and CD163, negative for S100, CD1a, and CD21. Liquid‐based comprehensive genomic profiling and tissue‐based cancer gene panel test identified *BRAF* ^V600E^ and *KRAS* ^G12R^ mutations with different variant allele fraction. Additional immunohistochemistry with an antibody specific to mutant *BRAF* ^V600E^ protein stained some proliferating histiocytes, consistent with ECD. Based on the genomic profiling results, we hypothesized that there was a coexistence of a clone harboring *BRAF* ^V600E^ and another clone harboring *KRAS* ^G12R^, and planned a combination therapy with BRAF and MEK inhibitors targeting each clone, respectively. The patient received oral encorafenib at 100 mg once daily and oral binimetinib at 15 mg twice daily. The combination therapy resulted in rapid resolution of symptoms and significant improvement in imaging findings.

**Conclusion:**

This case represents a unique presentation of ECD with concurrent *BRAF* ^V600E^ and *KRAS* ^G12R^ mutations. Combination therapy with encorafenib and binimetinib targeting each clone resulted in a remarkable therapeutic effect and was well‐tolerated. This is the first reported case of ECD treated with encorafenib and binimetinib. The combination therapy with BRAF and MEK inhibitors is one of the rational treatment options for cases of ECD with a suspicion of multiple clones.

**Abbreviations**
CDI: central diabetes insipidus
CGP: comprehensive genomic profiling
CT: computed tomography
ECD: Erdheim–Chester disease
FDA: Food and Drug Administration
FDG: fluorodeoxyglucose
LCH: Langerhans cell histiocytosis
MAP/ERK: mitogen‐activated protein kinase/extracellular signal‐regulated kinase
MRI: magnetic resonance imaging
PET: positron emission tomography
VAF: variant allele fraction

## 1 Introduction

Erdheim–Chester disease (ECD) is a rare form of non‐Langerhans cell histiocytosis with diverse clinical manifestations. Recent studies have shown that ECD is often associated with genomic mutations in the mitogen‐activated protein kinase/extracellular signal‐regulated kinase (MAPK/ERK) pathway, indicating that this disease is a clonal neoplastic disorder [1]. ECD is classified as a histiocytic neoplasm in the 5th edition of the World Health Organization classification of hematopoietic tumors [2]. Moreover, it is often challenging to diagnose ECD due to its diverse clinical presentation, equivocal histopathological findings, and difficulty in obtaining tissue samples. Genomic profiling, which can detect genomic abnormalities associated with ECD, is a valuable diagnostic tool, and molecular‐targeted therapy focusing on the identified driver mutations is promising.

*BRAF* ^V600E^ mutation is a well‐known gain‐of‐function mutation in the *BRAF* gene, resulting in the substitution of valine (V) with glutamic acid (E) at codon 600. This mutation leads to the constitutive activation of the BRAF protein, which activates the downstream MAPK/ERK signaling pathway, promoting uncontrolled cell proliferation. *BRAF* ^V600E^ mutation is implicated in various malignancies, including melanoma, colorectal cancer, and ECD, and is a critical target for therapeutic intervention [3, 4]. *KRAS* ^G12R^ mutation is also a gain‐of‐function mutation in the *KRAS* gene, in which glycine (G) at codon 12 is replaced by arginine (R). This alteration impairs the GTPase activity of the KRAS protein, leading to persistent activation of the KRAS signaling pathway. *KRAS* ^G12R^ mutation, like other *KRAS* mutations, drives oncogenesis by continuously transmitting growth signals within the cell and is frequently associated with various types of cancer [5, 6].

*BRAF* ^V600E^ is the most common driver mutation observed in over 50% of ECD cases [1], and the United States Food and Drug Administration (FDA) approved vemurafenib, a BRAF inhibitor, for *BRAF* ^V600E^‐mutant ECD in a Phase 2 trial [7]. The consensus guidelines for the treatment of ECD recommend BRAF inhibitors as the first‐line treatment for *BRAF* ^V600E^‐mutant ECD and recommend MEK inhibitors for ECD harboring another MAPK/ERK pathway mutation [1].

Herein, we report a rare case of a patient with ECD diagnosed with genomic profiling harboring both *BRAF* ^V600E^ and *KRAS* ^G12R^ mutations simultaneously and who well responded to the combination therapy with encorafenib, a BRAF inhibitor, and binimetinib, a MEK inhibitor. We obtained approval from our institutional tumor board and the administration board for using BRAF and MEK inhibitors. We also obtained informed consent from the patient for publication of this case report.

## 2 Case

**FIGURE 1:**
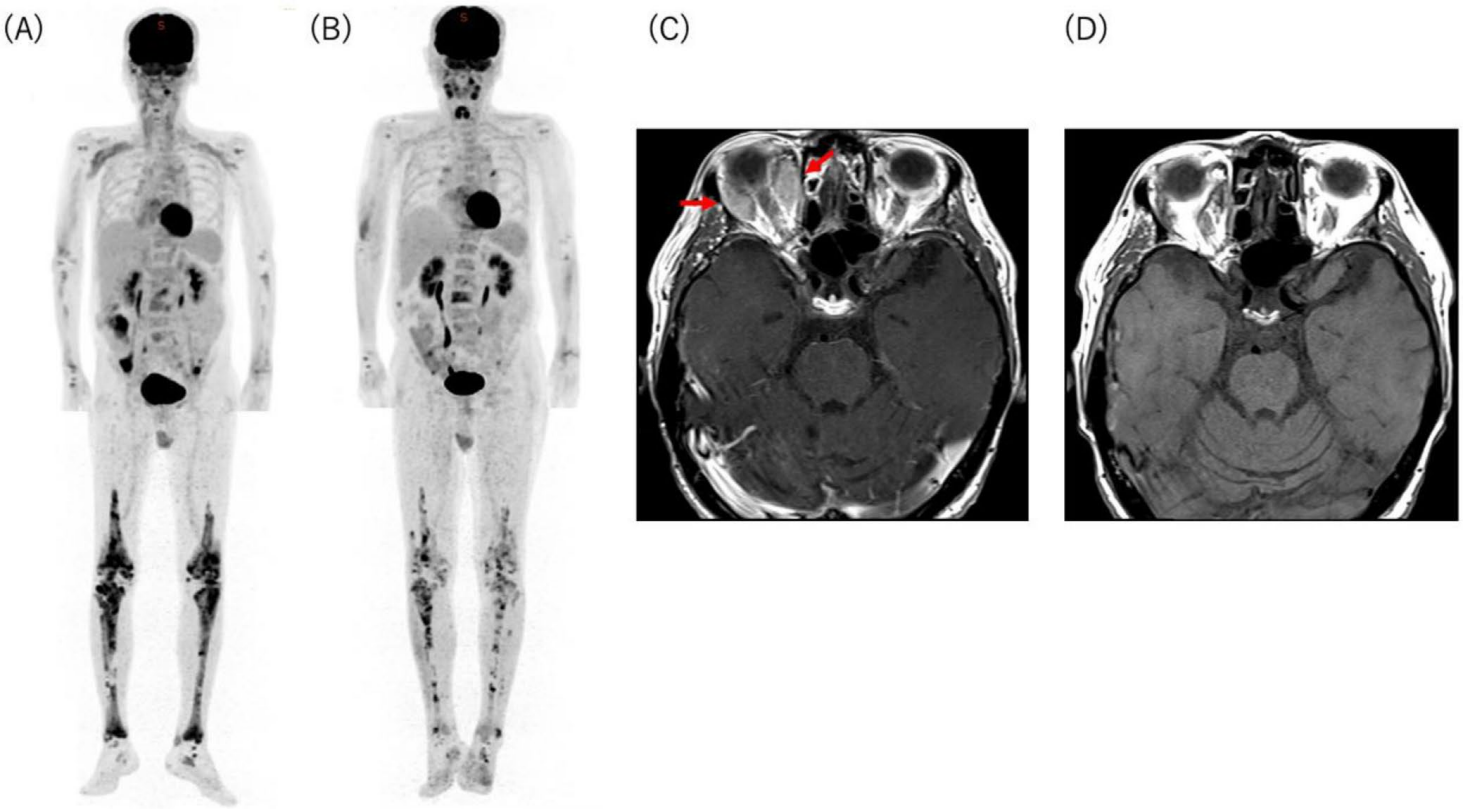
Imaging findings. (A) ^18^F‐FDG‐PET/CT scan images at diagnosis showing increased uptake in the mesentery, iliopsoas muscles, periaortic tissue, and bones. (B) PET/CT scan images 4 months after treatment initiation showing reduced uptake in these regions. (C) MRI scan images at diagnosis showing the right intraorbital lesion (arrow). (D) MRI scan images 6 months after treatment initiation showing a significant reduction in the size of the right intraorbital lesion. ^18^F‐FDG‐PET/CT, ^18^F‐fluorodeoxyglucose positron emission tomography/computed tomography; MRI, magnetic resonance imaging.

**FIGURE 2:**
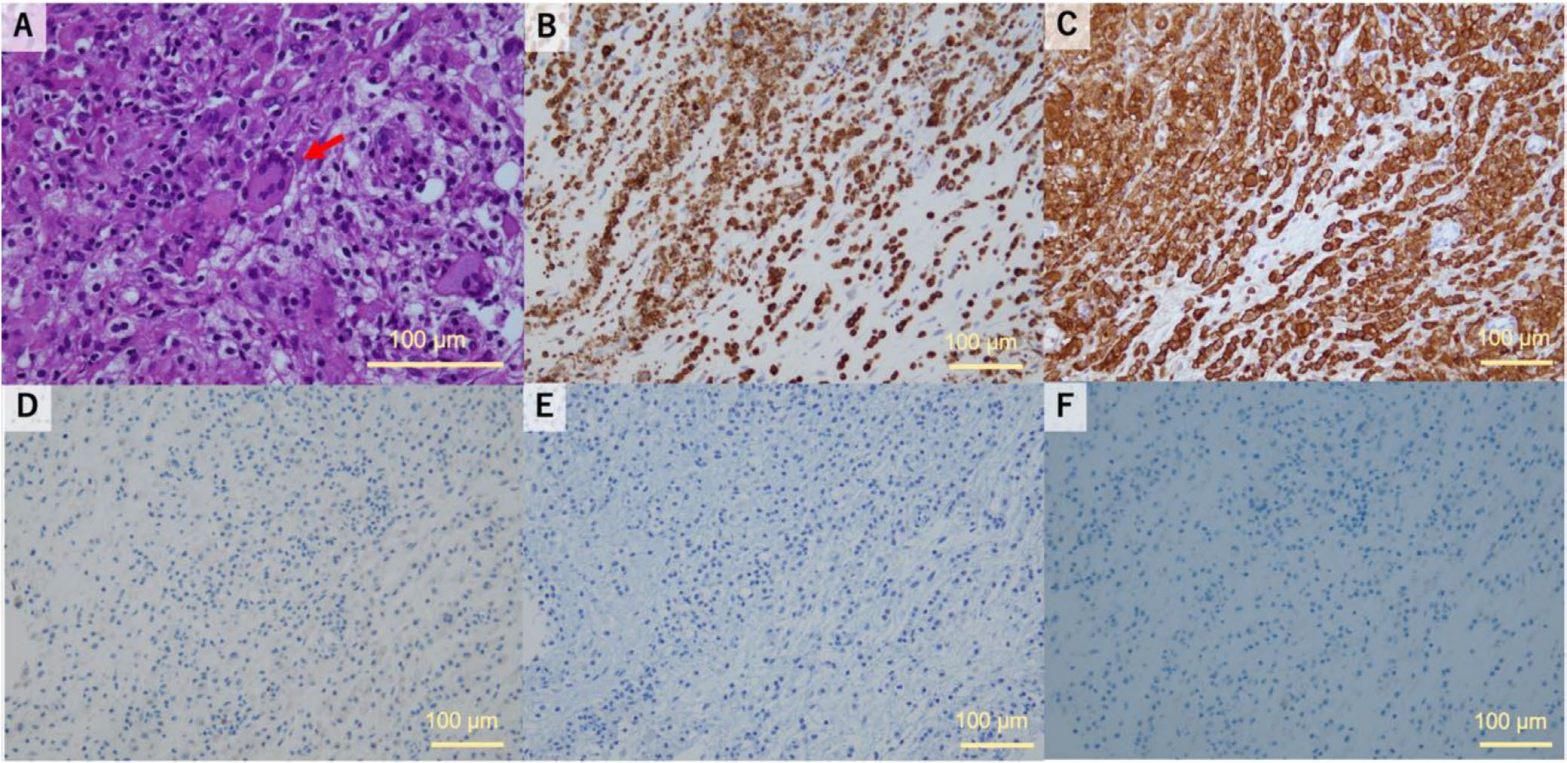
Pathological findings of the mesenteric lesion biopsy. (A) Hematoxylin and eosin staining shows multinucleated giant cells (arrow) and infiltration of foamy cells, which are positive for (B) CD68 and (C) CD163, and negative for (D) S100, (E) CD1a, and (F) CD21. Original magnification at image acquisition: ×200. Scale bar = 100 μm.

**FIGURE 3:**
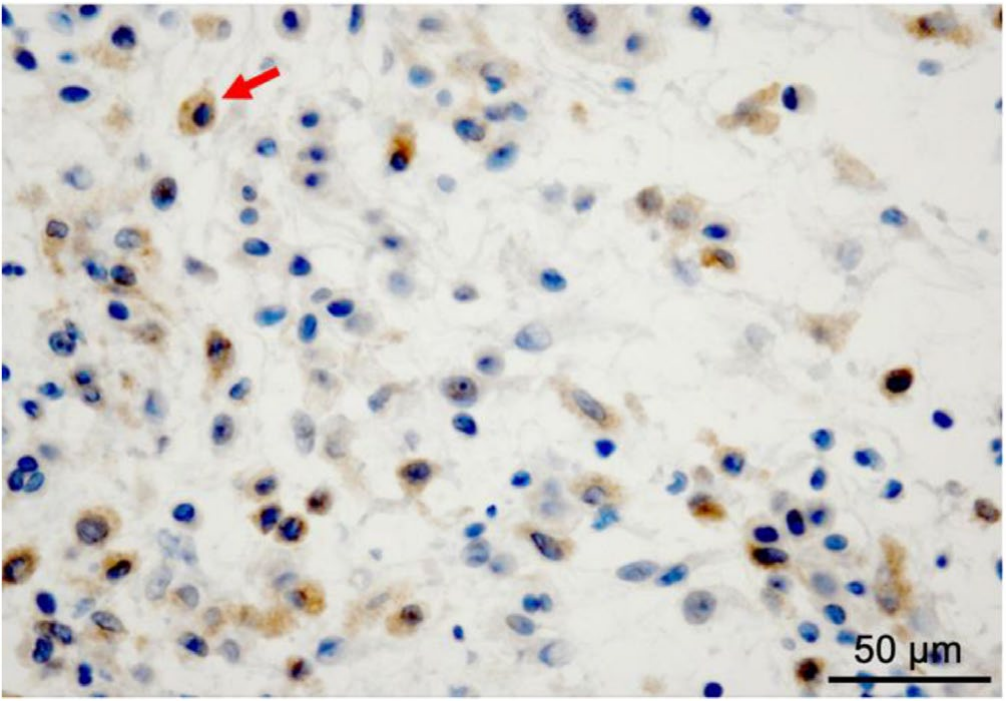
Additional pathological findings of the mesenteric lesion biopsy. Some histiocytes (arrow) are positive for *BRAF* ^V600E^. Original magnification at image acquisition: ×400. Scale bar = 50 μm.

A 70‐year‐old man was referred to Kanagawa Cancer Center in October 2022 with a mesenteric nodal lesion incidentally detected on computed tomography (CT) scan. He had a medical history of stroke (without sequelae) and dyslipidemia and had a long history of xanthelasma of the eyelids, dysarthria, ataxia, polydipsia, polyuria, and fatigue and had been experiencing diplopia for the past 6 months. A mesenteric lesion was detected on CT scan taken during the examination of a left distal radius fracture caused by a fall. Positron emission tomography (PET)/CT revealed ^18^F‐fluorodeoxyglucose (FDG) uptake in the mesentery, iliopsoas muscles, periaortic tissue, femurs, tibias, and pelvis (Figure 1A). Magnetic resonance imaging (MRI) of the brain revealed a right intraorbital lesion (Figure 1C) and T1‐weighted signal loss in the posterior pituitary lobe. After hypertonic saline infusion and desmopressin stimulation, the patient was diagnosed with central diabetes insipidus (CDI). We considered the right intraorbital lesion as the cause of his diplopia, whereas CDI was considered the cause of his polydipsia and polyuria. Given the possibility of malignancy, we performed a CT‐guided biopsy of the mesentery, which revealed infiltration of histiocytes positive for CD68 and CD163, and negative for S100, CD1a, and CD21 on immunohistochemistry, with unclear clonality (Figure 2). Initially, suspecting that the histiocytes were reactive cells associated with a tumor of an unknown primary site, we performed liquid‐based comprehensive genomic profiling (FoundationOne Liquid CDx), which identified *BRAF* ^V600E^ mutation with a variant allele fraction (VAF) of 1.83% and *KRAS* ^G12R^ mutation with a VAF of 19.78%. To exclude the possibility of histiocytosis harboring these genomic mutations, a tissue‐based cancer gene panel test (Ion AmpliSeq Cancer Hotspot Panel v2) was performed on the remaining tissue specimens, which identified *BRAF* ^V600E^ mutation with a VAF of 10.85% and *KRAS* ^G12R^ mutation with a VAF of 27.7% (Table 1). These results suggested that the histiocytes were tumor cells. Additional immunohistochemistry with an antibody specific to mutant BRAF^V600E^ protein stained some proliferating histiocytes (Figure 3). According to the pathological and genomic findings, along with the clinical and imaging features, we diagnosed the patient with ECD.

**TABLE 1:** Comparison of liquid‐based comprehensive genomic profiling and tissue‐based cancer gene panel test results.

Mutation	Liquid‐based profiling (VAF%)^a^	Tissue‐based profiling (VAF%)^b^
*BRAF* ^V600E^	1.83	10.85
*KRAS* ^G12R^	19.78	27.7

^a^ Variant allele fraction (VAF) detected in liquid‐based comprehensive genomic profiling (FoundationOne Liquid CDx).

^b^ VAF detected in tissue‐based cancer gene panel test (Ion AmpliSeq Cancer Hotspot Panel v2) from the mesenteric lesion.

Based on the genomic profiling results, we planned a combination therapy with BRAF and MEK inhibitors. Because no drugs had been approved for ECD in Japan, we obtained informed consent from the patient and approval from our institutional tumor board and the administration board for using BRAF and MEK inhibitors. The patient received oral encorafenib at 100 mg once daily (22% of the approved dose for melanoma) and oral binimetinib at 15 mg twice daily (33% of the approved dose for melanoma). After the initiation of treatment, the patient's fatigue and diplopia rapidly resolved. Improvement in FDG uptake in the mesentery and bilateral femurs and tibias was observed on PET/CT performed 4 months after treatment initiation, and a marked reduction in the size of the right intraorbital lesion was observed on brain MRI performed 6 months after treatment initiation (Figure 1B,D). The patient's overall condition improved significantly, and CDI was well controlled with oral desmopressin. Regarding the adverse events related to the treatment, swelling, and erythema of both eyelids were observed 3 weeks after starting treatment but improved with a 3‐day interruption of binimetinib. After the symptoms improved, binimetinib was resumed at the same dose, and no further adverse events were observed. After 21 months from the initiation of the treatment, his therapy was switched to oral dabrafenib at 150 mg twice daily and oral trametinib at 2 mg once daily because of the approval of reimbursement for *BRAF* mutation‐positive advanced solid tumors or recurrent solid tumors, including histiocytic disorders, in Japan. Although he experienced drug‐induced fever, it was well managed with antipyretics and dose adjustments. Currently, he is on an alternate‐day regimen with both dabrafenib and trametinib administered together on treatment days. Furthermore, he remains in remission 28 months after the initiation of combination therapy with BRAF and MEK inhibitors.

## 3 Discussion

In our ECD case, *BRAF* ^V600E^ and *KRAS* ^G12R^ mutations were concurrently detected before treatment. *BRAF* and *KRAS* mutations, which are driver mutations of oncogenes, participate in the same signaling pathway (MAPK/ERK pathway) and are usually mutually exclusive [8, 9]. The different VAF between *KRAS* ^G12R^ and *BRAF* ^V600E^ in both liquid‐based comprehensive genomic profiling (CGP) and tissue‐based cancer gene panel test in our case suggests the presence of separate clones harboring each mutation. Immunohistochemistry for BRAF^V600E^ protein further revealed the coexistence of BRAF^V600E^‐positive histiocytes and BRAF^V600E^‐negative histiocytes (Figure 3). Taken together, it was strongly inferred that in this case, there was coexistence of a clone harboring *BRAF* ^V600E^ and another clone harboring *KRAS* ^G12R^. To further investigate the hypothesis of the presence of two distinct clones, we considered performing a single‐cell mutation analysis using laser microdissection. However, the remaining tissue sample was insufficient. Furthermore, it is interesting to note that the VAF of *BRAF* ^V600E^ showed a significant discrepancy, being 1.83% in the liquid biopsy and 10.85% in the tissue biopsy, whereas for *KRAS* ^G12R^, the difference in VAF between the liquid and tissue samples was not prominent. Liquid‐based CGP effectively captures tumor heterogeneity, reflecting the characteristics of major clones throughout the body. Consequently, discrepancies may arise between the results of tissue‐based cancer gene panel test and liquid‐based CGP [10]. PET/CT findings indicate that ECD lesions were located throughout the body beyond the retroperitoneum, where the biopsy specimen was obtained (Figure 1A). It is possible that this case represents a heterogeneous tumor containing a higher proportion of *BRAF* ^V600E^ mutation‐negative clones, which may explain the lower VAF of the *BRAF* ^V600E^ mutation observed in the liquid‐based CGP.

Several studies have documented cases of ECD with concurrent *BRAF* ^V600E^ and *KRAS* mutations. ECD is often found alongside Langerhans cell histiocytosis (LCH), which is characterized by mutations in the MAPK/ERK pathway, including *KRAS* gene [1]. Notably, Nordmann et al. described a case where an acquired mutation, *KRAS* ^Q61H^, emerged in a new lesion that was negative for *BRAF* ^V600E^, during treatment with the BRAF inhibitor dabrafenib for ECD that harbored the *BRAF* ^V600E^ mutation [11]. In our case, the examination of tissue specimens did not indicate any findings suggestive of LCH, and both *BRAF* ^V600E^ and *KRAS* ^G12R^ mutations were identified concurrently before the initiation of treatment. To the best of our knowledge, this is the first reported case of ECD initially presenting with concurrent *BRAF* ^V600E^ and *KRAS* ^G12R^ mutations prior to treatment.

Regarding the targeted therapy for ECD with concurrent *BRAF* ^V600E^ and *KRAS* ^G12R^ mutations, the combination of a BRAF and MEK inhibitors, targeting downstream pathways of both BRAF and KRAS, resulted in a remarkable therapeutic effect in our case. Consensus guidelines recommend using BRAF inhibitors for cases with *BRAF* ^V600E^ mutation and MEK inhibitors for those with other MAPK/ERK pathway alterations [1]. There is limited data on the efficacy of MEK inhibitor monotherapy for *BRAF* ^V600E^‐mutant ECD and BRAF inhibitor monotherapy for *BRAF*‐wild‐type ECD [12]. A Phase 2 trial of the MEK inhibitor cobimetinib in histiocytic disorders did not achieve complete metabolic response in *BRAF* ^V600E^‐mutant ECD cases [13], suggesting that the depth of response was inferior compared to that of BRAF inhibitors. Additionally, multiple studies have reported the efficacy and safety of combination therapy with BRAF and MEK inhibitors for ECD [12, 14, 15]. Considering the potential for insufficient efficacy of monotherapy with either BRAF or MEK inhibitors in treating this exceptionally rare and rapidly progressing disease, we decided to introduce the combination therapy with both BRAF and MEK inhibitors as first‐line treatment. Based on the Phase 3 clinical trial, a combination therapy with encorafenib and binimetinib has been approved for the treatment of melanoma in the United States, Europe, and Japan [16]. While the FDA has approved vemurafenib for ECD, we selected encorafenib and binimetinib based on our experience with their use in melanoma. Although Wada et al. reported a case of *BRAF* ^V600*E*^‐mutant ECD successfully treated with encorafenib monotherapy [17], to date, there have been no reported cases of a combination therapy with encorafenib and binimetinib for the treatment of ECD.

Our treatment doses of encorafenib and binimetinib were well‐tolerated. We established the dosage for each drug based on previous studies indicating that patients with ECD require a significant reduction in therapeutic agents (25%–50% of the FDA‐approved dose for other indications) due to toxicity [15].

In conclusion, we experienced an exceptionally rare case of ECD initially presenting with concurrent *BRAF* ^V600E^ and *KRAS* ^G12R^ mutations. Combination therapy with encorafenib and binimetinib targeting each clone resulted in a remarkable therapeutic effect. The drugs were administered at significantly reduced doses from those approved for melanoma and were well‐tolerated. To the best of our knowledge, this is the first reported case of ECD treated with encorafenib and binimetinib. The combination therapy with BRAF and MEK inhibitors is one of the rational treatment options for cases of ECD with a suspicion of multiple clones.

## Author Contributions

**Yuto Hibino:** conceptualization, investigation, visualization, writing – original draft. **Rika Sakai:** conceptualization, supervision, writing – review and editing. **Hiroyuki Takahashi:** conceptualization, supervision, writing – review and editing. **Takaaki Takeda:** writing – review and editing. **Natsuki Hirose:** writing – review and editing. **Mayumi Tokunaga:** writing – review and editing. **Kota Washimi:** investigation, resources, writing – review and editing, visualization. **Tomoyuki Yokose:** investigation, resources, writing – review and editing. **Rika Kasajima:** investigation, resources, writing – review and editing. **Yukihiko Hiroshima:** investigation, resources, writing – review and editing, writing – original draft. **Yohei Miyagi:** investigation, resources, writing – review and editing, writing – original draft. **Hideaki Nakajima:** supervision, writing – review and editing.

## Ethics Statement

We obtained approval from our institutional tumor board and the administration board for using BRAF and MEK inhibitors.

## Consent

We obtained informed consent from the patient for publication of this case report.

## Conflicts of Interest

Yuto Hibino and Hiroyuki Takahashi received honoraria from Ono Pharmaceutical Co. Ltd.

## Data Availability

Data sharing not applicable to this article as no datasets were generated or analysed during the current study.
